# Supplementary material for: Chromothripsis during telomere crisis is independent of NHEJ, and consistent with a replicative origin
Source: Genome Res. 2019 May;29(5):737–49. doi: 10.1101/gr.240705.118 (PMC6499312; doi:10.1101/gr.240705.118)
Supplement: Supplemental Material [file supp_gr.240705.118_Supplemental_file_1.zip › contigs/annotated_contigs/DB37/contig.2.DB37_length_695_mean_cov_31.9884892086.docx]

**DB37_length_695_mean_cov_31.9884892086**

```
ATATTAGTGATAC|CTACGTCCGCTCGTTAATCTACACACATACATACGACGCTTTTCATACGTAAAATAAACGTCTTTATCGACGATC
              >chrUn_gl000220:103119-103742 - E=0e+00 p=0e+00
CCTTCTTTATAGAGCGATGTGTATTTATTTGTATAACACAGACACCTACATCTATCATACAGAAGTCTATTTCCATACAACCGATACGT

ATTTACCATACGCAAGAGTATTCAATGCAGAGATACACGTTGTCGTTGTTTGCATATAAGCGTACAGAAACGTTTACATTAATACATAT

AAGTAAACGCGTGGAAACGAAAGAAATAAAAAAGCGAAATGAGTCAACAGGCCGGGCACGGTGGCTCACGCCCGTCATCCCAGCACTTC

GAGAGGCCGAGGTGGGCGCATCACAGGAGGTCGGGAGTTGGAGACCAGCCTGAGCAACATGGAGAGACACGGCGTGCCTACTAAAAACA

CAAACATCAGCCAAGCCAGGCGTGGGGGTGCCTCCCTGTAATCCCCGCTAATCGGGAGGCTGAGGCAGGAGAAGCGCTCGAACCCGGGA

GGCGGAAGGTGCGGTGAGCCAAGACCGCGCCATTGCACTCTAGCCGTGGAAACAAGAGTGAAACTCTGTCTCAAAAGGACGAAACAGAA

AGAAAGAAAGA|AAG|GAAGGAAGGAAGGAAGGAAGGAAGGAAGAAAGAAAGAAAAGAAAGAAGGAA|TGAATGAA            >chr8:134982856-134982910 + E=1e-14
```
